# Filling the Gaps in a Fragmented Health Care System: Development of the Health and Welfare Information Portal (ZWIP)

**DOI:** 10.2196/resprot.1945

**Published:** 2012-09-19

**Authors:** Sarah HM Robben, Mirjam Huisjes, Theo van Achterberg, Sytse U Zuidema, Marcel GM Olde Rikkert, Henk J Schers, Maud M Heinen, René JF Melis, For The ZOWEL NN Study Group

**Affiliations:** 1 Department of Geriatric MedicineRadboud University Nijmegen Medical CentreNijmegenNetherlands; 2 Scientific Institute for Quality of HealthcareRadboud University Nijmegen Medical CentreNijmegenNetherlands; 3 Department of Primary CareUniversity of Groningen, University Medical Center GroningenGroningenNetherlands; 4 Department of Primary and Community CareCentre for Family Medicine, Geriatric Care and Public HealthRadboud University Nijmegen Medical CentreNijmegenNetherlands; 5 ZOWEL NNDepartment of Geriatric MedicineRadboud University Nijmegen Medical CentreNijmegenNetherlands

**Keywords:** Self-care, cooperative behavior, interdisciplinary communication, electronic health records, frail elderly

## Abstract

**Background:**

Current health care systems are not optimally designed to meet the needs of our aging populations. First, the fragmentation of care often results in discontinuity of care that can undermine the quality of care provided. Second, patient involvement in care decisions is not sufficiently facilitated.

**Objective:**

To describe the development and the content of a program aimed at: (1) facilitating self-management and shared decision making by frail older people and informal caregivers, and (2) reducing fragmentation of care by improving collaboration among professionals involved in the care of frail older people through a combined multidisciplinary electronic health record (EHR) and personal health record (PHR).

**Methods:**

We used intervention mapping to systematically develop our program in six consecutive steps. Throughout this development, the target populations (ie, professionals, frail older people, and informal caregivers) were involved extensively through their participation in semi-structured interviews and working groups.

**Results:**

We developed the Health and Welfare Information Portal (ZWIP), a personal, Internet-based conference table for multidisciplinary communication and information exchange for frail older people, their informal caregivers, and professionals. Further, we selected and developed methods for implementation of the program, which included an interdisciplinary educational course for professionals involved in the care of frail older people, and planned the evaluation of the program.

**Conclusions:**

This paper describes the successful development and the content of the ZWIP as well as the strategies developed for its implementation. Throughout the development, representatives of future users were involved extensively. Future studies will establish the effects of the ZWIP on self-management and shared decision making by frail older people as well as on collaboration among the professionals involved.

## Introduction

Current health care systems are not optimally designed to meet the needs of our aging populations [1]. First, they are characterized by fragmentation, which leads to inefficiency and can make health care efforts less effective [2,3]. Second, they do not facilitate the incorporation of patient perspectives in care decisions because they are designed according to a medical model that relies on care decisions being made by professionals with limited patient involvement [4].

Yet, the roles of patients and informal caregivers in our health care system are changing. Patients are now increasingly encouraged to become involved. There are several reasons for this. First, patients are involved in their care because it is they who make daily decisions about how they manage their disease (eg, they decide whether they take their medication or follow the lifestyle advice provided by professionals) [5]. Second, patient involvement is valued for moral and ethical reasons and considered a patient’s right [6]. Third, research has shown that increased patient involvement can have favorable effects, such as improved health outcomes and improved adherence [7-9]. Therefore, increasing the involvement of patients in their own care by enabling them to participate in decision making and by supporting them to manage their disease to the best of their ability is highly recommended.

However, increased patient involvement may be difficult to achieve in a health care system that suffers from fragmentation because both patients and professionals may already be struggling to meet the complex demands placed on them by such a health care system. In a fragmented health care system, care for a single patient, especially care for a frail older patient (an older patient suffering from a range of problems in the physical, psychological, and social domain), is often provided by multiple professionals who work in a variety of settings [1,10,11]. As a consequence, continuity of care (the degree to which a series of discrete health care events is experienced as coherent, connected, and consistent with the patient’s medical needs and personal context [11]), is limited. This undermines the quality of care provided [12,13]. Consequently, coordination of care across settings and services, by the sharing of accurate information between professionals and by the effective collaboration of professionals, patients, and informal caregivers, is badly needed [10,14,15].

Therefore, we developed a program aimed at: (1) facilitating self-management and shared decision making by frail older people, and (2) reducing fragmentation of care by enhancing collaboration among professionals involved in the care of frail older people through a multidisciplinary shared electronic health record (EHR) and personal health record (PHR). This paper describes the development of this program.

## Methods

The program, the Health and Welfare Information Portal (ZWIP), was initiated by ZOWEL NN, a collaborative of stakeholders in health care and welfare services, located in the city of Nijmegen, the Netherlands. The two main objectives for the program were: (1) to facilitate self-management and shared decision making by frail older people and their informal caregivers, and (2) to improve collaboration among professionals by enhancing and facilitating information sharing through a multidisciplinary shared EHR and PHR. Intervention mapping, a stepwise approach for the systematic development of theory- and evidence-informed interventions [16], was chosen as the method for developing the program. In the following sections, we will discuss the steps taken in this process. An overview is provided in Table 1.

**Table 1:** Overview of the intervention mapping process.

Steps	Methods	Results
1. Needs assessment	Problems analysis based on literature search; semi-structured interviews with frail older people and informal caregivers (n = 22); 2 meetings of working group of professionals (n = 15); and 1 meeting of working group of older people and informal caregivers (n = 4).	Logic model for self-management (Figure 1) and interprofessional collaboration (Figure 2).
2. Preparing matrices of performance objectives and determinants	Building matrices of performance objectives, determinants and change objectives based on the needs assessment.	Matrices of performance objectives and determinants for frail older people and informal caregivers, professionals, and the organizations of professionals (Appendices 1-3).
3. Selecting theory-informed intervention methods and practical strategies	Literature search for theories and methods and their effectiveness for the target populations; selection of theories and methods.	Theories used for the program: social cognitive theory (main theory), goal-setting theory, and elements of theories of organizational change.
		Methods and strategies used for professionals: modeling, active learning, direct experience, and creating facilitating conditions.
		Methods and strategies used for frail older people and informal caregivers: tailoring, modeling, guided practice, collaborative goal setting, and action planning.
4. Producing program components and materials	Requirements for Health and Welfare Information Portal (ZWIP) were defined in 3 additional meetings of working group of professionals (n = 15) and one additional meeting of working group of older people and informal caregivers (n = 4).	Main program component: the ZWIP.
	Subsequently, development of ZWIP in parallel with reviewing by working groups: 4 meetings of working group of professionals (n = 6); 3 meetings with two working groups of frail older people (n = 4).	Target population: frail older people ≥ 70 years, informal caregivers, and their professionals.
	Small pilot study of the ZWIP.	Setting: primary care.
		Materials: the ZWIP; bubble diagram and goal-setting forms; and personalized Internet-based and paper brochures with health promotion information concerning different domains of health, functioning, and well-being.
5. Planning program adoption, implementation, and sustainability	Program initiated by network of local stakeholders in health care and welfare services; future users involved extensively in development; necessity for health care system changes for frail older people felt at several levels (government, organizations, and professionals).	Implementation strategies for professionals: involvement in development; starting with early adopters; educational program (CME credits available) and e-learning; telephonic help desk available; coaching and e-coaching available; financial compensation; publicity and flyers; and incentives.
		Implementation strategies for employing organizations: financial compensation and educational program for employees.
		Implementation strategies for frail older people and informal caregivers: involvement in development, flyers, involvement of informal caregiver, involvement of family physician, Internet-based and paper version of the ZWIP, instruction in using the ZWIP by volunteer, and telephonic help desk available.
6. Planning for evaluation	Design of an evaluation plan.	Framework for process evaluation and evaluation of effects.

### Step 1: Needs Assessment

First, we assembled a planning group to develop the intervention. This planning group included the project manager, the project leader (RM), two researchers (SR and MHu), two family physicians, a geriatrician, a nurse scientist experienced in intervention mapping (MHe), an information technology consultant, and a long-term care facility physician.

This planning group analyzed the existing problems with self-management of frail older people and interprofessional collaboration in primary care. First, we performed a literature search for barriers to patient self-management and interprofessional collaboration. Second, we conducted semi-structured interviews at the homes of frail older people (n = 11) and informal caregivers (n = 11). They were invited to participate by their family physician or welfare organization and were purposively selected based on variation in living situation, socioeconomic position, and health and social problems. Interviewees were asked for their experiences with receiving information from health care and welfare professionals, informational continuity (ie, whether information concerning their health or well-being was exchanged between professionals), and interprofessional collaboration. Third, we established two working groups. The first group consisted of health care and welfare professionals (n = 15) who were involved in the care of frail older people. They were recruited through their employing organizations and were financially compensated for their time investments. Members included family physicians (n = 3), primary care nurses (n = 3), geriatricians (n = 2), municipality workers (n = 2), social workers (n = 2), a long-term care facility physician (n = 1), a pharmacist (n = 1), and a psychologist (n = 1). The second working group consisted of older people (n = 2) and informal caregivers (n = 2), who were asked to participate by older people participating in the user panel of ZOWEL NN. Both groups were asked to discuss the problems they experienced with self-management of frail older people and collaboration among professionals and they were asked to review and comment on the results from the literature search, semi-structured interviews, and the other working group.

Results of this needs assessment were integrated into a logic model. This model is derived from the Predisposing, Reinforcing, and Enabling Constructs in Educational/Environmental Diagnosis and Evaluation (PRECEDE) model [16,17] that displays behaviors, its consequences, and its determinants in a structured manner. As the problems described for each topic (self-management and collaboration) were too distinct to be compiled into one single logic model, we constructed a separate logic model for each program objective.

### Step 2: Preparing Matrices of Performance Objectives and Determinants

Based on the problem analysis, we defined performance objectives (ie, the behaviors required to achieve the program objectives) for each target population. These performance objectives were then crossed in matrices with those determinants of behavior that were known to have a major influence on behavior and were amenable to change. On the crossings of performance objectives and determinants, change objectives were formulated (ie, the highly specific outcomes the program should be aiming for). We designed these matrices for all target populations involved (ie, frail older people and their informal caregivers, professionals, and their employing organizations).

### Step 3: Selecting Theory-Informed Intervention Methods and Practical Strategies

We searched the literature for theories that were proven to be effective in changing the identified determinants or that were successfully used to enhance patient self-management or to promote collaboration among professionals. From these theories, we selected methods and strategies for our program. In this selection, we aimed for an optimal balance between the expected advances toward our program objectives and the investments required from the target populations.

### Step 4: Producing Program Components and Materials

Requirements for the program components were defined in additional meetings of the working groups of professionals and older people and informal caregivers. Subsequently, members of the planning group started development of program components. These components were reviewed by the working group of professionals and by two additional working groups of frail older people in an iterative process involving several rounds of reviewing by the working groups, the working groups making suggestions for improvement, and members of the planning group making adjustments. In this process, development and reviewing coincided, each working group being presented with the latest version of the components at the time of their meeting. Final versions of the program components were tested in a small pilot study involving two frail older people, two informal caregivers, and seven professionals.

### Step 5: Planning Program Adoption, Implementation, and Sustainability

A prerequisite for adoption and implementation of the program was met by the extensive involvement of the target population in its development and the commitment of the local collaborative of stakeholders in health care and welfare services. Further, implementation was facilitated by selecting implementation strategies that were tailored to the needs of each target population. Planning for sustainability was started early in the development of the program by searching for funding for incorporation of the program in everyday practice.

### Step 6: Planning for Evaluation

In this final step, we designed a plan for the evaluation of the program. This involved an evaluation of the effects of the program as well as a process evaluation.

## Results

### Step 1: Results of the Needs Assessment

**Figure 1:**
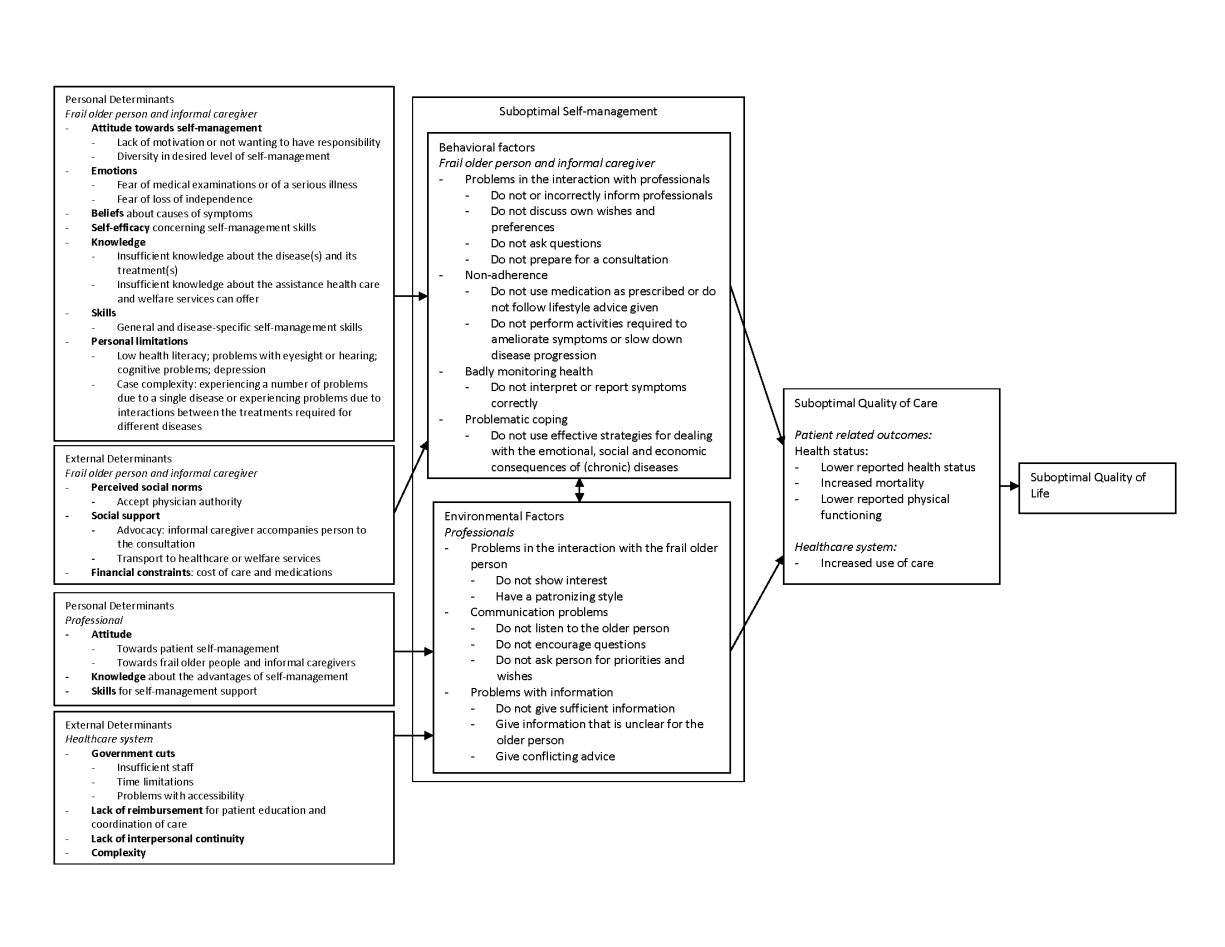
Logic model for self-management of frail older people.

**Figure 2:**
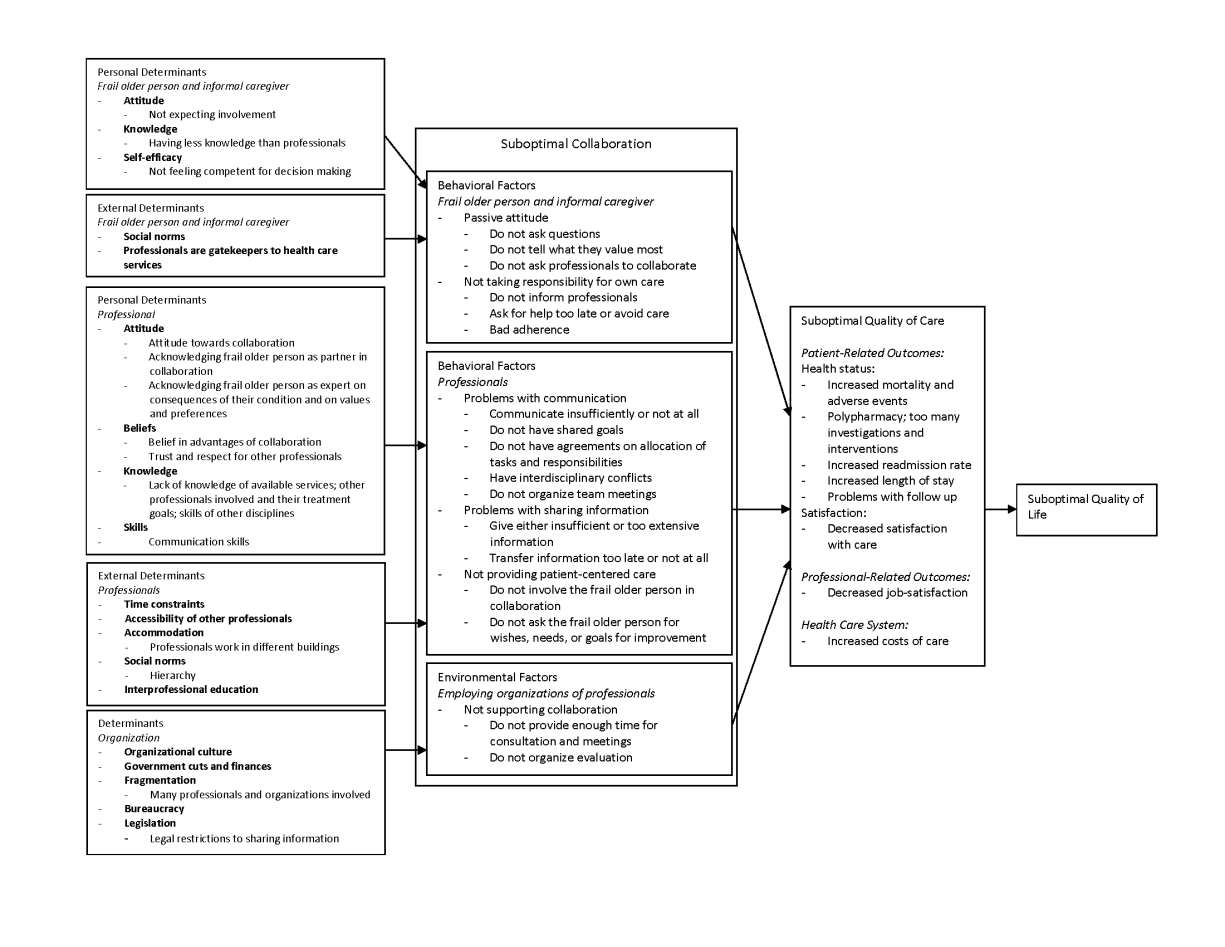
Logic model for collaboration among professionals.

An overview of the results of the needs assessment for self-management of frail older people is provided in the logic model shown in Figure 1 [5,7,13,18-34]. A second logic model representing collaboration among professionals is shown in Figure 2 [4,7,10,21,23,29-31,33-49]. Each logic model describes the problem (the last two columns), the behavioral and environmental factors that contribute to the problem (the second column), and the determinants that influence those factors (the first column). We will briefly discuss the results of the needs assessment in the following section. Knowledge of the Dutch health care system may help the interpretation of the results of this needs assessment; therefore, a summary of its characteristics is provided in Textbox 1 [50].

**Characteristics of the Dutch Health Care System.**

- All Dutch citizens are registered with their own family physician, usually over an extended period of time. This family physician functions as a gatekeeper; hospital care and specialist care (except for emergency care) can only be accessed with a referral by a family physician.
- When patients need other health care or welfare services (eg, home care, physiotherapy, or occupational therapy), they can generally choose between many providers offering these services.
- Funding of the Dutch health care system is organized by means of a compulsory social health insurance scheme.

#### Needs Assessment Concerning Frail Older People’s Involvement in Self-management

Frail older people, informal caregivers, professionals, and previous research reported problems with patient involvement in self-management. These problems related to frail older people and informal caregivers not performing the activities required, and professionals not encouraging or facilitating involvement.

Identified behaviors of frail older people and informal caregivers that contributed to these problems included: (1) not adequately informing professionals about their health situation nor asking sufficient questions [29,30], and (2) not adhering to medications prescribed or advice given [23,29,34]. These behaviors were influenced by many determinants such as attitude toward self-management because not all frail older people want to be involved extensively [7,33]; emotions such as fear of loss of independence [7,18]; self-efficacy for self-management [5,18,26,27]; knowledge about the disease, symptoms, and treatments [18,22,26]; skills [5,27]; personal limitations (eg, cognitive problems) [7,20,26,33]; perceived social norms [7,33]; social support, such as advocacy [7,18,26,27]; financial constraints [18,25,26]; and the high complexity of the health care system [34].

Important contributing behaviors of professionals were (1) not providing the frail older person with adequate information for self-management [20,26,34], and (2) not being genuinely interested in the frail older person and not encouraging questions [25,26,29]. Important determinants affecting these behaviors were attitude toward patient self-management [22,33], knowledge [22], skills for self-management support [20,22], and determinants related to the health care system [20,33].

#### Needs Assessment Concerning Collaboration Among Professionals

Professionals, patients, informal caregivers, and the literature cited problems with collaboration among professionals. The main behaviors that contributed to these problems were a lack of communication or insufficient communication [35,39,47]; delays in the transfer of information or information not being transferred at all [41,44]; giving either insufficient information (eg, not giving the information required by a particular discipline) [41,44] or too extensive information that was not read by professionals with already demanding work schedules; and not involving the frail older person in the collaboration between professionals. Important determinants influencing these behaviors included attitudes toward collaboration [42,45], beliefs in the advantages of collaboration [45], knowledge about the information needed by other disciplines [45], communication skills [35,42,45], and external factors such as time constraints [35] and legal restrictions to the sharing of information [45]. However, for professionals in the working groups, more practical determinants were the most important, such as not knowing which other professionals were involved in the care of the frail older person, not knowing them personally [39,40,42,48], and not being able to contact these professionals (eg, due to part-time work or busy telephone lines) [35,39,40].

### Step 2: Results on Matrices of Performance Objectives and Determinants

Based on our needs assessment, we defined performance objectives for both program objectives and for each target population involved (Appendices 1 and 2). Also, we reviewed the determinants shown in Figures 1 [5,7,13,18-34] and 2 [4,7,10,21,23,29-31,33-49] in order to select those determinants of behavior that were considered both important to target and modifiable. For the first program objective, aimed at facilitating self-management, we developed two matrices: one for frail older people and informal caregivers and one for professionals. For frail older people and informal caregivers, targeted determinants were attitudes, skills and self-efficacy, knowledge, and social support. For professionals, targeted determinants were attitudes, knowledge, skills, and organization. For the second program objective, aimed at enhancing collaboration, we designed three matrices: one for professionals, one for their organizations, and one for frail older people and informal caregivers. For professionals, targeted determinants were attitudes and beliefs, knowledge, skills, and accessibility; for their organizations, the targeted determinant was organizational culture; and for frail older people and informal caregivers, targeted determinants were attitude, self-efficacy, knowledge, skills, social norms and social support, and accessibility. We then crossed the performance objectives with these determinants to design matrices of change objectives. For example, for the performance objective “professional communicates with other professionals involved” and the determinant “knowledge,” a change objective was “professional states that problems in communication lead to adverse outcomes for frail older people.” Therefore, we wanted our program to increase professionals’ knowledge about the effects of communication problems. Appendix 3 provides an example of a matrix of change objectives.

### Step 3: Selected Theories, Methods, and Strategies

Social cognitive theory [51] was selected as the main theory behind the program because it has been successfully used in the past for interventions aimed at improving patient self-management and in Internet-based interventions focusing on improving self-management [52-54]. A key concept of social cognitive theory is perceived self-efficacy: the beliefs people have about their capabilities to produce the effects they desire by their own actions [55]. If self-efficacy is low, people are less likely to either act or to continue trying when facing difficulties [51]. We included several methods and strategies derived from this theory in the program, based on their ability to change the targeted determinants of behavior. For professionals, we included active learning, direct experience, modeling, and facilitation. For frail older people and their informal caregivers, we included modeling, guided practice, and tailoring. Further, elements of goal-setting theory [56] (ie, goal setting and action planning) [57] were included in the program to assist frail older people and informal caregivers in describing what is most important to them, to help them to achieve their goals, and to increase their involvement in the care process. Goal-setting theory highlights the importance of setting specific, difficult goals because people who set such goals perform better that those who are merely asked to do their best [56]. Last, we incorporated elements of several theories of organizational change into the program. Methods used from these theories were providing training and coaching, and creating facilitating conditions [16,58].

### Step 4: Characteristics of ZWIP

Taking the former steps of the intervention mapping process into account, we developed the main component of the program: the ZWIP. The ZWIP is a personal, Internet-based conference table for multidisciplinary communication and information exchange for frail older people, their informal caregivers, and professionals. It can be considered to be both a shared EHR and PHR. The ZWIP is aimed at frail older people identified through a specific screening method and includes: (1) a tool for multidisciplinary communication in a secure environment that enables communication through sending messages between the frail older person, informal caregiver, and the professionals involved; (2) an overview of health care and welfare professionals involved in the care of the frail older person and their contact information; (3) information about the frail older person’s health, functioning, and social situation as well as the care provided; (4) the goals and action plans of the frail older person and the informal caregiver, which are formulated with them during home visits by nurses or social workers by means of a goal-setting tool; and (5) tailored educational materials for the frail older person and informal caregiver. Fundamental to the ZWIP is the central position of the frail older person, who can view the information included and who decides which professionals are granted access to his personal ZWIP. As a rule, messages that are communicated within the ZWIP are visible for all professionals with access to the ZWIP as well as for the frail older person and informal caregiver. This allows everyone concerned to remain informed about the frail older person’s situation and enables everyone to bring up their own relevant observations in an ongoing conversation. However, at the request of frail older people and professionals, we also included the option of sending a private message to an individual person.

After development, as a final step before implementation, we conducted a small pilot study of the ZWIP. The most important lessons learned from this pilot were practical issues such as the need to communicate as unambiguously as possible.

### Step 5: ZWIP Program Adoption and Implementation

Strategies used for the adoption and implementation of the program were tailored to the needs of each particular target population. We will describe the main strategies used in the next paragraphs; an overview of all strategies is provided in Table 1 (step 5).

For health care and welfare professionals, our most important strategy was an interdisciplinary educational program for health care and welfare professionals involved in the care of frail older people. This program consisted of 3 three-hour meetings concerning the following subjects: (1) the concept of frailty and identification of frailty, as this was required to identify the frail older people who were the program’s target population; (2) providing self-management support to frail older people by thoroughly informing them and using collaborative goal setting; (3) interdisciplinary collaboration, including information about what each discipline has to offer in the care for frail older people; and (4) working with the ZWIP. Except for its educational content, the educational program also served as a method for identifying and bringing together local health care and welfare professionals involved in the care of frail older people because the program enabled professionals to get acquainted with each other. The educational meetings were held near local family practices and all local professionals working with frail older people were invited to participate. Another important strategy was that we aimed to ensure the participation of intrinsically motivated early adopters. Further, we tailored the implementation of the program to each setting by providing family medical practices with several options for implementation, which allowed them to choose the method that would best meet their local needs and circumstances. Also, we provided financial compensation for time invested in the program, gave coaching and e-coaching in using the ZWIP, and had a telephonic help desk available.

For frail older people and informal caregivers, we had two main strategies. First, we involved their family physician in the project, who actively promoted their participation. Second, we aimed to either facilitate the use of information technology or to make the use of information technology by frail older people redundant, as we were aware that they often have low computer literacy. Hence, we provided them with an Internet-based version of the ZWIP as well as a paper version of the ZWIP, which held all information that was included in the Internet-based ZWIP except for the communication; we offered them a home visit by a volunteer, who could either demonstrate the ZWIP to inform them about its possibilities or could train them in using the ZWIP themselves; and we had a telephonic help desk available during office hours.

### Step 6: Preparing for Evaluation of the ZWIP

As a final step in the intervention mapping process, we planned the evaluation of the ZWIP. This evaluation will involve both a process evaluation and an effect evaluation. In the process evaluation, we will evaluate the implementation of the intervention, exposure of the target populations to the intervention, experiences of the target populations with the intervention, and barriers and facilitators to the use of the intervention. This will be studied using a combination of quantitative and qualitative data (ie, surveys, data about both the use of the ZWIP and exposure to its implementation strategies, and semi-structured interviews). The effects of the ZWIP program will be evaluated by means of a controlled clinical trial. Outcome measures will be the effects of the program on interprofessional collaboration, patient self-management and autonomy, patient outcomes such as functioning and quality of life, and use of care. Also, cost-effectiveness of the ZWIP will be evaluated. Last, as we consider the interprofessional educational program an important part of the implementation, the effects of this program on interprofessional collaboration will be evaluated separately. This will be done in a before-and-after study using several validated questionnaires (ie, the Attitudes Toward Health Care Teams Scale [59], the Interprofessional Attitudes Questionnaire [60,61], and the Team Skills Scale [62]) followed by semi-structured interviews with purposively selected participants.

## Discussion

This paper describes the successful development of a program aimed at facilitating self-management and shared decision making by frail older people and their informal caregivers and at reducing fragmentation of care through improving collaboration among professionals. For this development, the intervention mapping framework was used and future users were involved extensively. In the past, this framework has also been successfully used for the development of health promotion programs aimed at such diverse topics as leg ulcers [63], physical activity of employees in sedentary occupations [64], sexually transmitted disease, pregnancy and human immunodeficiency virus prevention [65], and asthma self-management [66]. To our knowledge, this is the first time that intervention mapping was successfully used to develop an intervention that specifically targets collaboration between professionals.

A major advantage of the use of intervention mapping was that it facilitated the systematic incorporation of the needs and preferences of the target population as well as evidence from previous research. We can exemplify this with our first program objective, which concerned self-management and shared decision making. Previous research has shown that most older people prefer a less active role in medical decision making [67], but they do want to be informed, and they want their concerns and wishes to be taken into account when decisions are made [7]. Still, there is enormous variation in the extent to which older people wish to participate in decision making [7]. Therefore, we designed our program to meet the basic level of involvement wanted by most older patients (eg, by providing information about their health and customized educational materials; by including goal setting to gain knowledge of their goals and preferences; and by educating professionals in self-management support), yet made the program flexible to more extensive patient involvement in decision making (eg, by incorporating action planning for patients willing to engage in it and by facilitating patients’ communication with professionals).

Further, the program benefitted from the involvement of the target populations because they brought up a wide range of knowledge and perspectives [16]. Moreover, the target populations were able to specify which problems found in the literature were considered most pressing by members of their own population because they were highly knowledgeable of their characteristics and circumstances. For example, although we initially assumed that lack of continuity of information was an important barrier to collaboration, the involvement of the working group of professionals demonstrated that more basic obstacles to collaboration existed (ie, practical problems concerning communication, such as not knowing which other professionals are involved or not being able to contact them due to differing working hours). Therefore, we decided to shift focus of the program to include facilitation of communication as well. This enabled designing a program that was tailored to meet their needs, thereby increasing the chances of an effective intervention and a successful implementation.

Although involvement of the target population was considered important, it also presented a challenge. First, involving frail older people proved to be difficult. For the limited number of frail participants in the working groups, problems such as not being able to attend the meetings due to health problems limited their ability to participate. Therefore, we also invited older people who were not frail to join the working groups. Also, for some of the frail older people participating in the semi-structured interviews, cognitive problems made it difficult for them to express their views about the rather abstract interview topics. Therefore, although frail older people were involved in the development process, their involvement was less than we would have preferred. Second, the evidence gathered from previous research and the different working groups did not always point in the same direction. An example was the discussion about whether or not all messages should be visible to everyone with access to the ZWIP. The working group of professionals was hesitant at first to make all messages visible, and the working groups of frail older people were divided. In the end, both groups mentioned that there were instances in which they felt a private message was absolutely required. In such cases, the planning group made a final decision. These decisions were made based on a thorough deliberation on all the arguments available from the literature and the working groups as well as arguments concerning feasibility.

Although the ZWIP is a systematically developed evidence-informed intervention, its future success depends highly on its successful implementation and its use by professionals in everyday practice. Implementation and use will be monitored and adaptations will be made whenever required. Further, future use of the ZWIP in everyday practice will have to establish the added value of the communication tool of ZWIP in relation to already existing communication methods.

In summary, this article describes the successful development of the ZWIP, a personal, Internet-based conference table for multidisciplinary communication and information exchange for frail older people, their informal caregivers, and professionals. We expect that the ZWIP will be able to increase the involvement of frail older people and informal caregivers in their care and will improve collaboration among professionals. Therefore, we expect that the ZWIP will contribute to filling the gaps in our fragmented health care systems.

## Multimedia Appendix 1

Performance objectives for each target population related to self-management.

## Multimedia Appendix 2

Performance objectives for each target population related to collaboration.

## Multimedia Appendix 3

Section of matrix of change objectives on enhancing collaboration of professionals.

## Abbreviations

EHR: electronic health record
PHR: personal health record
ZWIP: Health and Welfare Information Portal
