# Supplementary material for: Filling the Gaps in a Fragmented Health Care System: Development of the Health and Welfare Information Portal (ZWIP)
Source: JMIR Res Protoc. 2012 Sep 19;1(2):e10. doi: 10.2196/resprot.1945 (PMC3626145; doi:10.2196/resprot.1945)
Supplement: Supplementary file 1 [file resprot_v1i2e10_app1.pdf]

**Appendix 1.** Performance objectives for each target population related to self-management

| Performance objectives (PO) | |
|---|---|
| | *Frail older person and informal caregiver…* |
| PO.1.1. | Monitors health status |
| PO.1.2. | Responds adequately to changes in health status |
| PO.2.1. | Interacts with healthcare and welfare professionals |
| PO.2.2. | Participates actively in problem solving |
| PO.2.3. | Participates actively in designing a treatment plan aimed at maintaining and improving health |
| PO.2.4. | Participates actively in the development of specific action plans targeting parts of the treatment plan |
| PO.3.1. | Participates in effectuating the treatment plan that has been agreed upon with professional |
| PO.4.1. | Deals adequately with disease, limitations and treatment |
| PO.4.2. | Uses supportive services in the community |
| PO.4.3. | Copes effectively with the emotional and psychological consequences of disease |
| | *Professional…* |
| PO.1. | Builds up an adequate patient-caregiver relationship with the frail older person |
| PO.2. | Underlines the central role the patient has in caring for him- or herself |
| PO.3.1. | Assesses the assumptions the frail older person has about his/her diseases |
| PO.3.2. | Assesses the knowledge the frail older person has about his/her diseases |
| PO.3.3. | Assesses what activities the frail older person already performs to self-manage his/her diseases |
| PO.4.1. | Provides the frail older person with customized information about his/her chronic conditions, which agrees with his health condition and the information he/she already has |
| PO.4.2. | Teaches the frail older person skills for monitoring and interpreting symptoms |
| PO.5.1. | Encourages the frail older person to be active in the management of his/her own diseases |
| PO.5.2. | Collaborates with the frail older person to make shared-decisions about the care plan |
| PO.6.1. | Agrees on a plan for follow-up with the older person |
| PO.6.2. | Provides ongoing follow-up |
