# Supplementary material for: Filling the Gaps in a Fragmented Health Care System: Development of the Health and Welfare Information Portal (ZWIP)
Source: JMIR Res Protoc. 2012 Sep 19;1(2):e10. doi: 10.2196/resprot.1945 (PMC3626145; doi:10.2196/resprot.1945)
Supplement: Supplementary file 2 [file resprot_v1i2e10_app2.pdf]

Appendix 2. Performance objectives for each target population related to collaboration

Performance objectives (PO)	
	Professional…
PO.1.	Shares relevant information with other professionals
PO.1.1	Asks client permission for sharing of information
PO.2.	Communicates with other professionals involved
PO. 2.1.	Communicates regularly and effectively
PO.2.2.	Clarifies the roles and responsibilities other professionals have
PO. 2.3.	Asks other professionals for their treatment goals and discusses own treatment goals
PO.3.	Involves client in collaboration
PO.3.1.	Asks client for wishes and goals and discusses these
PO. 3.2	Gives priority to client's goals in care plan and discusses other goals
	Frail older person and informal caregiver…
PO.1.	Contacts professionals when necessary
PO.2.	Gives professionals permission to exchange information about him/herself
PO.3.	Asks professionals involved to consult each other
PO.4.	Discusses goals for care plan with professional
PO.5.	Aims to achieve goals of care plan
	Organization…
PO.1.	Facilitates collaboration
PO.1.1.	Facilitates the communication of staff with professionals outside the organization
PO.1.2	Evaluates the results of employees' collaboration with professionals from different organizations
