# Supplementary material for: Filling the Gaps in a Fragmented Health Care System: Development of the Health and Welfare Information Portal (ZWIP)
Source: JMIR Res Protoc. 2012 Sep 19;1(2):e10. doi: 10.2196/resprot.1945 (PMC3626145; doi:10.2196/resprot.1945)
Supplement: Supplementary file 3 [file resprot_v1i2e10_app3.pdf]

**Appendix 3.** Section of matrix of change objectives on enhancing collaboration of professionals

| Professional… | Attitudes and Beliefs | Knowledge | Skills | Accessibility |
|---|---|---|---|---|
| PO.2. Communicates with other professionals involved | AB.2.a. Describes that a single discipline is not able to meet all the healthcare and well-being related needs of a particular frail older person<br><br>AB.2.b. Expresses the conviction that communicating with other professionals involved improves the quality of care delivered<br><br>AB.2.c. Expresses respect for, and trust in the other professionals involved<br><br>AB.2.d. Expresses the conviction that the involvement of other professionals improves the care provided to, and the well-being of frail older people | K.2.a. States that teams that are not collaborating produce worse health outcomes in frail older people, and lower satisfaction by clients and professionals at increased costs<br><br>K.2.b. States that problems in communication lead to adverse outcomes for frail older people<br><br>K.2.c. Describes which professionals are involved in the care of a particular frail older person<br><br>K.2.d. Describes how and when other professionals involved can be contacted | S.2.a. Demonstrates ability to collaborate<br><br>S.2.b. Demonstrates ability to access available database for the up-to-date address and telephone numbers of other professionals involved<br><br>S.2.c. Demonstrates ability to keep own address and telephone number in database up-to-date<br><br>S.2.d. Demonstrates using different methods for communication | A.2. Other professionals involved are available for consultation |
| PO. 2.1. Communicates regularly and effectively | AB.2.1. Expresses the conviction that the benefits of communication outweigh the time investments required for communication | K.2.1.a. States that communication improves by having regular face-to-face contact<br><br>K.2.1.b. States that better communication leads to better health outcomes for frail older people | S.2.1.a. Demonstrates the ability to listen and to provide own perspective<br><br>S.2.1.b. Demonstrates the ability to negotiate in case of disagreements and to reach consensus<br><br>S.2.1.c. Demonstrates the ability to adjust language to the person spoken to | A.2.1. Other professionals involved are available for consultation |

| | | | | |
|---|---|---|---|---|
| | | | S.2.1.d. Demonstrates the ability to evaluate own activities<br>S.2.1.e. Demonstrates the ability to give constructive feedback and to deal appropriately with feedback given by others<br>S.2.1.f. Demonstrates the ability to resolve conflicts | |
| PO.2.2. Discusses the sharing of roles and responsibilities with other professionals involved | AB.2.2.a. Expresses the importance of clarity about the allocation of tasks<br>AB.2.2.b. Expresses the importance of respecting the roles other professionals have | K.2.2.a. Describes the roles, skills and expertise of other disciplines involved<br>K.2.2.b. Describes how care tasks can be delegated | S.2.2.a. Demonstrates the ability to discuss the sharing of roles and responsibilities<br>S.2.2.b. Demonstrates the ability to delegate, share and transfer tasks in the care of a frail older person | A.2.2. Other professionals involved are available for consultation |
| PO. 2.3. Asks other professionals what their treatment goals are and discusses own treatment goals | AB.2.3. Describes the goals other professionals have as important | K.2.3. States the treatment goals other professionals involved have | S.2.3. Demonstrates the ability to write down own care goals in database | A.2.3. Other professionals involved are available for consultation |
